# Effect of Empagliflozin on Whole Body, Cardiac, and Renal Sympathetic Outflows in Type 2 Diabetes

**DOI:** 10.1016/j.jacadv.2025.102304

**Published:** 2025-11-08

**Authors:** Anne T. Reutens, Dulari Hakamuwa Lekamlage, Agus Salim, Jonathan E. Shaw, Murray D. Esler, Mark E. Cooper, David M. Kaye

**Affiliations:** a Clinical Diabetes and Epidemiology, Baker Heart and Diabetes Institute, Melbourne, Victoria, Australia; b Diabetes and Population Health, Baker Heart and Diabetes Institute, Melbourne, Victoria, Australia; c Centre for Epidemiology and Biostatistics, Melbourne School of Population and Global Health, The University of Melbourne, Melbourne, Victoria, Australia; d The Institute for Mental and Physical Health and Clinical Translation (IMPACT), Deakin University, Geelong, Victoria, Australia; e School of Mathematics and Statistics, The University of Melbourne, Melbourne, Victoria, Australia; f School of Public Health and Preventive Medicine, Monash University, Melbourne, Victoria, Australia; g Human Neurotransmitter Laboratory, Baker Heart and Diabetes Institute, Melbourne, Victoria, Australia; h Department of Cardiology, Alfred Hospital, Melbourne, Victoria, Australia; i Department of Diabetes, Monash University, Central Clinical School, Melbourne, Victoria, Australia; j Heart Failure Research Group, Baker Heart and Diabetes Institute, Melbourne, Victoria, Australia; k Monash Alfred Baker Centre for Cardiovascular Research, Monash University, Melbourne, Victoria, Australia

**Keywords:** empagliflozin, hemodynamics, norepinephrine spillover kinetics right heart catheter, SGLT2 inhibitor, sympathetic nervous activity

## Abstract

**Background:**

Sodium glucose co-transporter type 2 inhibitors improve cardiovascular outcomes in people with type 2 diabetes, though mechanisms by which this occurs remain unclear.

**Objectives:**

The purpose of this study was to evaluate empagliflozin’s effect on whole body, cardiac, and renal sympathetic activity in people with type 2 diabetes and cardiovascular disease or high cardiovascular risk.

**Methods:**

Adults with type 2 diabetes, randomized to 25 mg/day empagliflozin or placebo for 12 weeks, had norepinephrine (NE) spillover kinetics assessed during right heart catheterization. Endpoints were mean spillover rates at the end of treatment, adjusted for baseline (primary outcome whole-body spillover; secondary outcomes cardiac and renal spillovers).

**Results:**

Of 15 participants randomized to empagliflozin (15 completed treatment) and 17 to placebo (16 completed treatment), most were Caucasian males, mean age 67.9 years, glycated hemoglobin 8.0%, and body mass index 31.1 kg/m^2^. Baseline NE spillover rates did not differ between the 2 groups. Twelve weeks of empagliflozin treatment did not significantly alter whole body, cardiac, or renal NE spillover rates compared to placebo (*P* > 0.30 for all comparisons). Empagliflozin significantly lowered pulmonary capillary wedge pressure (adjusted between-group difference: −1.8 mm Hg; 95% CI: –3.4 to −0.1), and glycated hemoglobin (−0.3%; 95% CI: −0.6 to −0.02), with trends toward lower diastolic blood pressure (−3.9 mm Hg; 95% CI: −7.7 to −0.1) and body weight (−2.6 kg; 95% CI: −3.8 to −1.4), with no impact on heart rate or N-terminal pro-B-type natriuretic peptide.

**Conclusions:**

In this exploratory study, 12 weeks of empagliflozin treatment lowered cardiac filling pressures, but without the anticipated reflex increase in sympathetic nervous activity.

Long-term outcome trials with sodium glucose co-transporter type 2 inhibitors (SGLT2i) have shown impressive benefits in primary and secondary prevention of cardiovascular disease,^1^, ^2^, ^3^, ^4^ especially heart failure,^5^, ^6^, ^7^, ^8^ and lowering the risk of progressive kidney disease^9^ in people with diabetes. While SGLT2 inhibitors were originally developed to treat hyperglycemia, glycated hemoglobin (HbA1c) reduction is relatively modest and is not the major mediator of the cardiovascular benefit, as demonstrated through mediation analysis^10^ and meta-regression analysis^11^ of the cardiovascular outcome trials, and mediation analysis of cardiovascular disease outcomes in genetic variants causing SGLT2 inhibition.^12^

Several alternative mechanisms have been proposed for the beneficial cardiorenal effects of SGLT2i, including osmotic diuresis, improved cardiac fuel from ketones,^13^ decreased inflammation and oxidative stress,^14^ or changes in epicardial fat.^15^ The clinical observation that SGLT2i reduced blood pressure but did not increase heart rate suggested attenuation of sympathetic nervous activity (SNA) by this class of drugs.^16^^,^^17^ Chronic sympathetic nervous system (SNS) overactivity is postulated to cause myocyte hypertrophy and fibrosis leading to left ventricular remodeling^18^; renal arterial and venous vasoconstriction leading to salt and water retention, and peripheral neurogenic vasoconstriction leading to vascular hypertrophy.^19^ This suggests that blocking SNS overactivity may improve cardiovascular function. For example, beta-blockers are now considered essential for treatment of patients with heart failure with reduced left ventricular ejection fraction, based on considerable clinical trial evidence.^20^^,^^21^

SGLT2i reduced directly recorded renal SNA in hypertensive rats^22^ and diabetic rabbits.^22^^,^^23^ In human studies using muscle SNA (MSNA), there was no change in this or in heart rate after 4 days of treatment with SGLT2i, at the time of maximum diuresis, despite reduced blood pressure and weight.^24^ Twelve weeks of empagliflozin treatment reduced MSNA in people with type 2 diabetes with and without heart failure.^25^ However, in another study comparing the effect of an SGLT2i with hydrochlorothiazide in patients with type 2 diabetes, there was no change in MSNA with either drug after 6 weeks of treatment.^26^

In order to obtain a more nuanced, organ-specific assessment of SNA, invasive testing with blood sampling is required to measure the release of norepinephrine (NE) into the bloodstream, or “spillover,” by that organ (Central Illustration). Accordingly, we conducted this study to evaluate the effect on whole body, cardiac, and renal SNA, assessed by NE spillover kinetics, of 12 weeks of oral empagliflozin 25 mg taken daily compared to matching placebo in adults with type 2 diabetes and cardiovascular disease. The dose of empagliflozin used in this study, selected so that the maximum response would be elicited in the endpoints, corresponded to the higher dose arm of the EMPA-REG (Empagliflozin Cardiovascular Outcome Event Trial in Type 2 Diabetes Mellitus Patients–Removing Excess Glucose) study^1^ and was higher than the dose used in the previously mentioned heart failure trials (which was 10 mg daily).^6^^,^^7^

## Methods

### Participants

We enrolled adults (aged 18-85 years) with type 2 diabetes who were on stable diabetes therapy. Key inclusion criteria were HbA1c 6.5% to 10.0% (48-86 mmol/mol) at screening, with a body mass index 20 to 40 kg/m^2^ and an estimated glomerular filtration rate (eGFR) ≥30 mL/min/1.73 m^2^. Eligible participants had high cardiovascular risk, defined as a confirmed history of coronary artery disease, or age ≥30 years with documented symptomatic atherosclerotic noncoronary cardiovascular events, or age ≥50 years with 2 or more cardiovascular disease risk factors (diabetes for ≥10 years, hypertension or on blood pressure–lowering treatment, dyslipidemia or on treatment for it, albuminuria).

The key exclusion criteria were type 1 diabetes, a previous ketosis history, uncontrolled hyperglycemia, NYHA functional class III or IV heart failure, an acute cardiovascular disease event within the previous 2 months, cardiomyopathy, moderate-to-severe valvular heart disease, or current smoking status. The full eligibility criteria are provided in Supplemental Appendix 1.

### Study design

EMPA-SNS (Effect of empagliflozin on whole body, cardiac, and renal sympathetic outflows in type 2 diabetes) was a prospective, phase 4, randomized, double-blind, placebo-controlled parallel-group study, conducted at the Baker Heart and Diabetes Institute, Melbourne, Australia (Australian New Zealand Clinical Trial Registry ACTRN12617000490370). The study was performed between April 9, 2018, and December 14, 2022. The Alfred Hospital Ethics Committee approved the study protocol. All participants gave written informed consent. The study was conducted in accordance with the International Conference on Harmonization Good Clinical Practice guidelines and the Declaration of Helsinki.

After screening, participants underwent baseline SNA testing. Whole body, cardiac, and renal NE spillover studies were undertaken with samples obtained at right heart catheterization (RHC), during which hemodynamic indices were measured.

**Figure 1:**
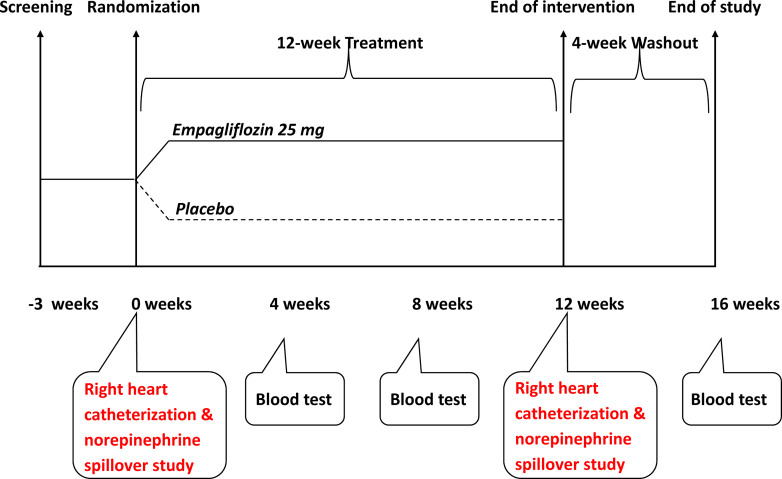
**Study Design for EMPA-SNS Trial** Schematic diagram summarizing the study visits and the tests done at each visit. EMPA-SNS = Effect of empagliflozin on whole body, cardiac, and renal sympathetic outflows in type 2 diabetes.

Eligible participants were then randomized to empagliflozin or placebo in a 1:1 ratio. Randomization was carried out centrally using random permuted block randomization. The participants received either 25 mg empagliflozin or matching placebo daily. Participants were telephoned at 1 week and 2 weeks to record adverse events (AEs) and assess glucose levels. They were then reviewed at 4 weeks and 8 weeks for recording of AEs, vital signs and weight, and blood tests to assess renal function and full blood count. At 12 weeks, the baseline investigations were repeated. There was a further follow-up visit at 16 weeks; 4 weeks after discontinuation of the study drug (Figure 1). Data collection and analyses were performed blinded to treatment allocation. Study drug compliance was assessed by pill count. Satisfactory compliance was defined as adherence to study medication ≥80% and ≤120%.

### WHOLE BODY, CARDIAC, AND RENAL NOREPINEPHRINE SPILLOVER STUDIES AND RHC

Sympathetic activity was assessed by isotope dilution during an infusion of tritiated NE to measure NE spillover kinetics for the whole body and regionally for the heart and kidney.^27^, ^28^, ^29^ Participants had a light and very low carbohydrate breakfast on the morning of the catheterization with no tea, coffee, or caffeine-containing foods or beverages. Breakfast was eaten at least 3 hours before the scheduled catheterization time. Central venous catheter placement was performed under local anesthesia via a 6F percutaneous introducing sheath inserted usually via the brachial vein or sometimes via the right jugular vein.

Central venous catheterization was performed under fluoroscopic control. Resting systolic and diastolic pulmonary arterial pressures, mean pulmonary artery pressure, mean right atrial pressure, and pulmonary capillary wedge pressure (PCWP) were measured at end-expiration as the average of ≥3 cardiac cycles at rest. The coronary sinus and right renal vein were then sequentially catheterized under fluoroscopic control to obtain venous blood samples. Arterial blood samples were obtained simultaneously from the radial artery. Cardiac output was measured by thermodilution.

Throughout the course of the catheter studies levo-[7-3H]-NA (specific activity of 11-25 Ci/mmol [New England Nuclear]) was infused intravenously for the assessment of cardiac, renal and whole-body NE spillover as previously described.^29^, ^30^, ^31^ Renal plasma flow was estimated by measuring the rate of plasma clearance of para-aminohippuric acid (Dalichi-Sankyo). Coronary sinus plasma flow measurements used in earlier research^32^^,^^33^ were not available here. The measure of cardiac sympathetic activity was the coronary sinus-arterial plasma NE concentration difference, adjusted for 3H-NE tracer extraction across the heart,^34^ that is:

(CS–A)+(E×A)pg/ml

where CS is coronary sinus plasma NE concentration; A is arterial plasma NE concentration; E is the fractional extraction of plasma 3H-NE in transit through the heart. All plasma neurochemical determinations were made using high-performance liquid chromatography with electrochemical detection. Timed collection of effluent and subsequent liquid scintillation counting assessed the radioactivity in plasma.

### Study endpoints

The primary efficacy endpoint was mean whole body spillover rate at the end of treatment adjusted for baseline. Key secondary endpoints were mean cardiac sympathetic activity and mean renal spillover rate, at the end of treatment adjusted for baseline.

Additional endpoints were PCWP, systolic and diastolic office blood pressures, HbA1c, fasting plasma glucose, and 24-hour urinary sodium, glucose, and albumin excretion.

Key safety outcomes were the incidence of serious AEs and AEs, mean body weight and body mass index at the end of treatment adjusted for baseline.

### Sample size justification and study power

The protocol initially planned for 17 participants per group, which would give this study power of >80% with an α = 0.05 and 2-sided hypothesis testing, to demonstrate group differences of ≥10% in log NE concentration. Because of COVID-19 pandemic lockdowns in 2020 to 2021, the number of participants required to be randomized was reduced to 16 participants per group, which would provide power >78%, α = 0.05, and 2-sided.

### Statistical analysis

The primary analysis was an intention-to-treat analysis using data from all randomized participants. Per-protocol analyses were also reported. Statistical analyses were conducted using Stata, version 15.1 (Stata) and R, Version 4.0.3 (R Foundation for Statistical Computing), at a 2-sided 0.05 level of significance. *P* values were not adjusted for multiplicity.

Standardized mean differences were used to compare baseline covariates between empagliflozin and control participants. A standardized mean difference of 0.1 (10%) denoted a significant imbalance in the baseline covariate. The primary and secondary efficacy analyses were conducted using analysis of covariance models. Missing data were handled by multiple imputation. Model diagnostics performed to evaluate model assumptions identified influential observations in whole body (2 participants), renal (2 participants), and cardiac (3 participants) data. Therefore, to mitigate the effects of these outliers, robust linear regression was used instead of the ordinary linear regression for all the NE spillover endpoints and those other secondary endpoints which violated model assumptions. Ordinary linear regression was used for analyzing the remaining endpoints. Unless stated otherwise, results are presented as mean (SD).

## Results

### Trial population

**Figure 2:**
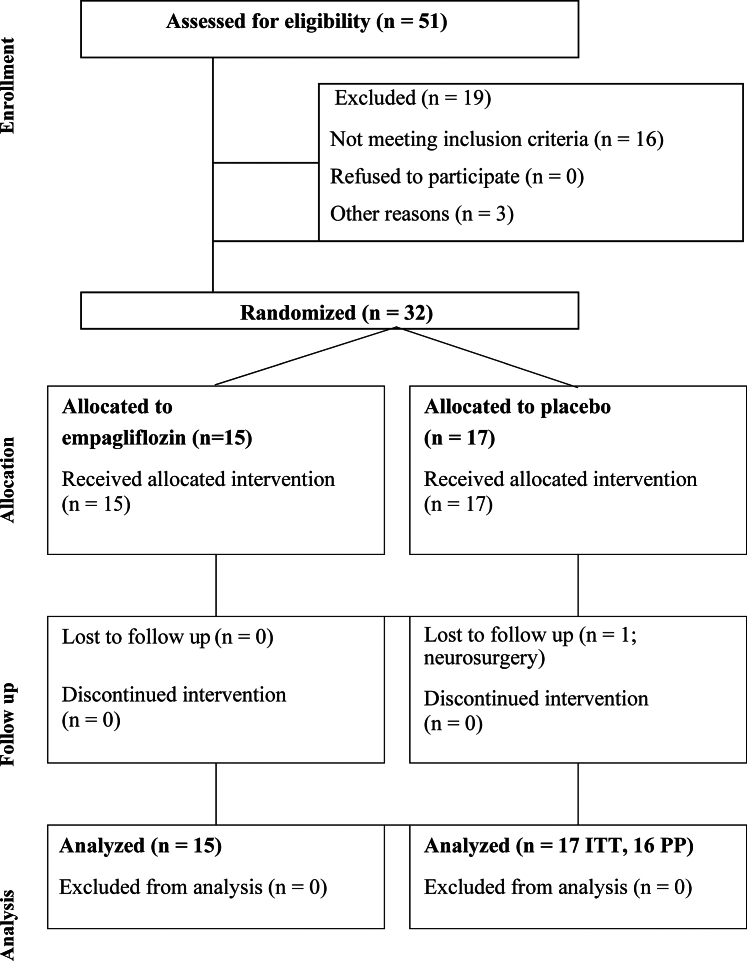
**Flowchart for Participants in This Study** This diagram describes each phase of the EMPA-SNS trial, from enrollment and treatment allocation, through follow-up, to data analysis. Abbreviation as in Figure 1.

Participant disposition is shown in Figure 2. We screened 51 participants and randomized 15 participants to the empagliflozin arm and 17 to the placebo arm. These 32 formed the intention-to-treat population, of which 15 and 16 participants in the empagliflozin and placebo control arms, respectively, completed the treatment period. These 31 formed the per-protocol population.

Baseline characteristics are summarized in Table 1. Participant demographics were comparable between the 2 groups. 65.6% were male, 71.9% were Caucasian. The mean (SD) age was 67.9 (5.4) years, and the mean body mass index was 31.2 (4.2) kg/m^2^. We did not have enough (statistical) evidence to conclude that the 2 groups were different in whole body NE spillover rates or renal NE spillover rates were between the 2 arms. Participants randomized to take empagliflozin, when compared to those assigned to placebo, had a baseline cardiac sympathetic activity that was ∼66 ng/min higher, and higher HbA1c, office systolic and diastolic blood pressures, and eGFR (see Table 1).

**Table 1:** Baseline Characteristics of Enrolled Participants According to Randomization

	Empagliflozin (n = 15)	Placebo (n = 17)	Overall (N = 32)	Standardized Mean Difference
Age, y	69.0 (6.3)	66.9 (4.5)	67.9 (5.4)	0.39
Female, n (%)	5 (33)	6 (35)	11 (34)	
Ethnicity, n (%)				
Caucasian	11 (73)	12 (70)	23 (72)	
Asian	1 (7)	1 (6)	2 (6)	**0.09**
Others	3 (20)	4 (24)	7 (22)	
Clinical characteristics				
Weight, kg	87.4 (12.7)	88.1 (14.7)	87.7 (13.6)	**0.05**
BMI, kg/m^2^	30.9 (4.2)	31.4 (4.3)	31.1 (4.2)	0.12
Office SBP, mm Hg	139.5 (11.2)	135.5 (8.2)	137.4 (9.8)	0.399
Office DBP, mm Hg	77.3 (9.3)	76.2 (7.2)	76.7 (8.2)	0.133
Pulse rate, beats/min	76.7 (13.9)	75.0 (8.5)	75.8 (11.2)	0.144
Cardiovascular disease history, n (%)				
History of coronary artery disease				
Medical treatment only	0	1 (5.88)	1 (3.13)	0.354
History of myocardial infarction	2 (13.33)	2 (11.76)	4 (12.5)	0.047
Coronary artery bypass graft	2 (13.33)	0	2 (6.25)	0.555
Percutaneous coronary intervention	0	3 (17.64)	3 (9.38)	0.655
History of cerebrovascular disease	0	1 (5.88)	1 (3.13)	0.354
History of peripheral artery disease	0	0	0	
Glucose-lowering therapy, n (%)				
Medication taken alone or in combination				
Metformin	13 (86.67)	17 (100)	30 (93.75)	0.55
Insulin	7 (46.67)	4 (23.53)	11 (34.38)	0.50
Median daily dose, IU	71.5	63.5	71.5	
Sulfonylurea	6 (40)	8 (47.06)	14 (43.75)	0.14
Dipeptidyl peptidase-4 inhibitor	7 (46.67)	5 (29.41)	12 (37.5)	0.36
Thiazolidinedione	0	0	0	
Glucagon-like peptide-1 receptor agonist	1 (6.67)	6 (35.29)	7 (21.88)	0.75
Antihypertensive therapy, n (%)				
Angiotensin-converting enzyme inhibitors/angiotensin receptor blockers	13 (86.67)	14 (82.35)	27 (84.38)	0.12
Beta-blockers	5 (33.33)	2 (11.76)	7 (21.88)	0.53
Diuretics	3 (20)	5 (29.41)	8 (25)	0.22
Calcium-channel blockers	7 (46.67)	8 (47.06)	15 (46.88)	0.01
Mineralocorticoid receptor antagonists	1 (6.67)		1 (3.13)	0.38
Moxonidine	1 (6.67)	1 (5.88)	2 (6.25)	0.03
Lipid-lowering therapy, n (%)				
Statins	12 (80)	17 (100)	29 (90.63)	0.71
Fibrates	0	1 (5.88)	1 (3.13)	0.35
Ezetimibe	2 (13.33)	1 (5.88)	3 (9.38)	0.25
Cholestyramine	0	1 (5.88)	1 (3.13)	0.35
Anticoagulants/antiplatelets, n (%)				
Acetylsalicylic acid	8 (53.33)	4 (23.53)	12 (37.5)	0.64
Clopidogrel (P2Y12 inhibitor)		2 (11.76)	2 (6.25)	0.52
Dipyridamole 3 inhibitor	1 (6.67)	0	1 (3.13)	0.38
Factor Xa inhibitors	1 (6.67)	0	1 (3.13)	0.38
Direct thrombin inhibitor	1 (6.67)	0	1 (3.13)	0.38
Laboratory results				
HbA1c, %	7.9 (0.7)	8.1 (0.8)	8.0 (0.7)	0.26
Insulin, mUnits/L	11.9 (8.4, 19.5)	18.1 (10.4, 26.9)	15.0 (10.1, 22.3)	0.557
Total cholesterol, mmol/L	3.6 (0.6)	3.6 (0.6)	3.6 (0.6)	**0.05**
Triglycerides, mmol/L	1.6 (1.1, 1.8)	1.40 (1.2, 1.9)	1.45 (1.2, 1.9)	0.28
eGFR, mL/min per 1.73 m^2^	79.5 (70.0, 99.0)	76.0 (68.0, 83.5)	90.0 (76.0, 99.0)	0.82
NT-proBNP, ng/L	190.4 (317.1)	63.3 (51.6)	122.9 (225.7)	0.56
Hemoglobin, g/L	138.3 (12.8)	137.1 (8.0)	137.1 (10.3)	0.11
Hematocrit, %	0.4 (0.03)	0.4 (0.02)	0.4 (0.03)	0.14
Urine ACR, mg/mmol	7.9 (17.6)	4.4 (9.4)	6.0 (13.7)	0.245
24-h urinary volume, ml (SD)	1,618.7 (632.1)	1,592.2 (560.5)	1,604.6 (585.5)	0.044
24-h urinary glucose, mmol	28.2 (43.3)	39.1 (65.6)	34.0 (55.7)	0.195
24-h urinary sodium, mmol	160.9 (86.6)	147.8 (64.0)	153.9 (74.5)	0.172
Catheterization characteristics				
PAS, mm Hg	23.0 (22.0, 26.5)	23.0 (21.5, 30.2)	23.0 (21.5, 29.0)	0.12
PAD, mm Hg	9.6 (3.1)	9.7 (4.2)	9.6 (3.7)	**0.024**
PA mean, mm Hg	15.60 (2.9)	16.1 (4.5)	15.8 (3.7)	0.123
PCWP, mm Hg	7.9 (3.2)	8.1 (4.3)	8.0 (3.7)	**0.07**
Right atrial pressure, mm Hg	3.40 (1.80)	4.12 (2.78)	3.77 (2.35)	0.31
Whole body spillover rate, ng/min	704.6 (383.6)	719.7 (372.1)	712.4 (371.4)	**0.04**
Renal spillover rate, ng/min	200.2 (85.3)	211.1 (146.3)	205.4 (116.6)	**0.09**
Cardiac spillover rate, ng/min	434.5 (225.1)	368.2 (226.5)	400.3 (224.5)	0.29

Data shown are mean (SD), or median (25th, 75th percentile), unless otherwise stated.

ACR = albumin to creatinine ratio; BMI = body mass index; DBP = diastolic blood pressure; eGFR = estimated glomerular filtration rate; HbA1c = glycated hemoglobin; NT-proBNP = N-terminal pro-B-type natriuretic peptide; PA = pulmonary artery pressure; PAD = pulmonary artery diastolic pressure; PAS = pulmonary artery systolic pressure; PCWP = pulmonary capillary wedge pressure; SBP = systolic blood pressure.

A standardized mean difference of <0.1 indicates balance between the groups.

### Norepinephrine spillover outcomes

There were no significant differences between the empagliflozin and control groups at 12 weeks in regard to whole-body spillover rate (primary outcome) or to renal NE spillover rate or cardiac sympathetic activity (Table 2). Similar results were achieved in the per protocol analysis.

**Table 2:** Changes in Main Endpoint Parameters Between Baseline and 12 Weeks

	Empagliflozin		Placebo		Estimated Treatment Difference at 12 Weeks Adjusted for Baseline, Empagliflozin Vs Placebo	*P* Value
	Baseline	12 Weeks	Baseline	12 Weeks	Estimate (95% CI)	
	Mean (SD)	Mean (SD)	Mean (SD)	Mean (SD)		
Whole body spillover rate	704.6 (383.6)	717.2 (324.4)	719.7 (372.1)	626.1 (449.4)	124.1 (−125.3 to 373.5)	0.34
Renal spillover rate	200.2 (85.3)	214.7 (122.3)	211.1 (146.3)	204.4 (177.4)	22.9 (−52.2 to 98.1)	0.556
Cardiac spillover rate	434.5 (225.1)	378.5 (208.5)	368.2 (226.5)	404.5 (356.7)	11.6 (−86.2 to 109.4)	0.818
Weight, kg	87.4 (12.7)	83.9 (12.1)	88.1 (14.7)	87.3 (14.6)	−2.6 (−3.8 to −1.4)	**4.00E-04**
Office SBP, mm Hg	139.5 (11.25)	126.5 (12.2)	135.5 (8.3)	131.0 (9.0)	−3.9 (−7.7 to −0.1)	0.055
Office DBP, mm Hg	77.3 (9.3)	70.4 (7.8)	76.2 (7.1)	73.4 (7.0)	−1.5 (−6.4 to −3.4)	0.552
Pulse rate, beats/min	76.7 (13.9)	71.3 (9.0)	75.0 (8.5)	72.3 (8.1)	−1.2 (−23.6 to −21.2)	0.918
HbA1c, %	7.9 (0.7)	7.4 (0.4)	8.1 (0.8)	7.8 (0.8)	−0.3 (−0.6 to −0.02)	**0.043**
Insulin, mUnits/L	14.6 (8.6)	12.6 (7.3)	20.3 (11.7)	17.2 (12.2)	−8.9 (−28.0 to 10.2)	0.372
Total cholesterol, mmol/L	3.6 (0.6)	3.9 (0.6)	3.6 (0.6)	3.7 (0.7)	0.17 (−0.2 to 0.5)	0.364
Triglycerides, mmol/L	1.5 (0.5)	1.6 (0.7)	1.7 (0.9)	1.7 (0.7)	0.01 (−0.3 to 0.3)	0.918
eGFR, mL/min per 1.73m^2^	75.5 (15.0)	71.7 (19.1)	87.1 (13.1)	87.0 (11.6)	−5.5 (−12.4 to 1.3)	0.127
NT-proBNP, ng/L	190.4 (317.1)	231.5 (384.1)	63.3 (51.6)	70.7 (46.5)	−8.863 (−28.0 to 10.2)	0.372
Hemoglobin, g/L	138.3 (12.8)	138.7 (13.0)	137.12 (8.0)	135.1 (8.3)	2.461 (−2.1 to 7.0)	0.298
Hematocrit, %	0.4 (0.03)	0.4 (0.03)	0.4 (0.00)	0.4 (0.02)	0.019 (0.005–0.033)	**0.018**
Urine ACR, mg/mmol	7.9 (17.6)	6.2 (10.8)	4.41 (9.4)	5.4 (11.8)	−0.7 (−1.7 to 0.3)	0.172
24-h urinary volume, ml	1,618.7 (632.1)	2,248.4 (730.9)	1,592.2 (560.5)	1,729.6 (692.1)	446.2 (44.9–847.6)	**0.038**
24-h urinary glucose, mmol	28.2 (43.3)	480.3 (248.6)	39.1 (65.6)	31.5 (52.5)	453.1 (330.9–575.4)	**8.24E-08**
24-h urinary sodium, mmol	160.9 (86.6)	164.0 (65.0)	147.8 (64.0)	147.1 (69.0)	8.86 (−26.6 to 44.3)	0.628
PAS, mm Hg	25.5 (6.6)	22.7 (5.6)	24.8 (5.7)	25.4 (4.2)	−3.2 (−5.6 to −0.9)	**0.011**
PAD, mm Hg	9.6 (3.1)	7.3 (2.4)	9.7 (4.2)	9.0 (3.2)	−1.6 (−3.0 to −0.3)	**0.025**
PA mean, mm Hg	15.6 (2.9)	13.4 (2.9)	16.1 (4.5)	15.7 (3.3)	−2.0 (−3.5 to −0.5)	**0.015**
PCWP, mm Hg	7.9 (3.2)	5.5 (2.5)	8.11 (4.3)	7.4 (3.2)	−1.8 (−3.4 to −0.1)	**0.048**
RAP, mm Hg	3.4 (1.8)	3.0 (2.2)	4.1 (2.8)	4.4 (2.5)	−0.966 (−2.5 to 0.5)	0.226

These data are from the intention-to-treat analysis.

RAP = right atrial pressure; other abbreviations as in Table 1.

### Other secondary outcomes

As anticipated, the empagliflozin-treated participants had significantly lower HbA1c and weight, and higher urinary glucose, adjusted for baseline, compared to those on placebo (Table 2). There was no difference in urinary sodium concentration assessed at the end of 12 weeks of treatment. Twenty-four-hour urine volumes were higher in the empagliflozin-treated group compared to placebo, when adjusted for baseline (∼466 mL; 95% CI: 45-847; *P* = 0.038). The office systolic blood pressures were no different between the groups when adjusted for baseline, but there was a trend toward a lower diastolic blood pressure in those who received empagliflozin (intention-to-treat analysis −3.9 mm Hg; 95% CI: −7.7 to −0.1; *P* = 0.055). There was no difference in heart rate. From the RHCs, pulmonary arterial pressure indices and PCWP were significantly lower in those receiving empagliflozin compared to those on placebo, when adjusted for baseline (pulmonary artery mean −2.0 mm Hg; 95% CI: −3.5 to −0.5; *P* = 0.015; PCWP -1.8 mm Hg; 95% CI: −3.4 to −0.1; *P* = 0.048). Red blood cell parameters were significantly increased in the empagliflozin-treated participants (Table 2, Supplemental Table 1).

**Figure 3:**
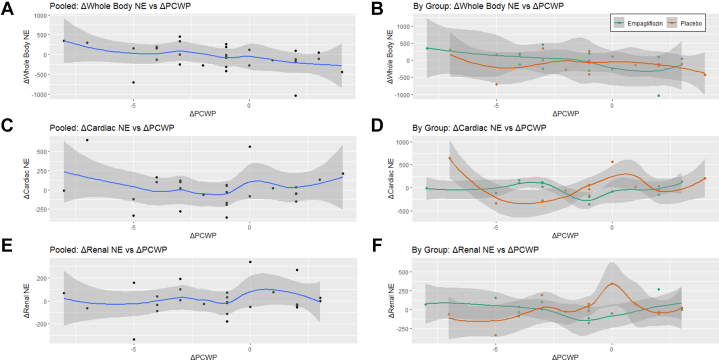
**Change in Sympathetic Nervous Activity Vs Change in Pulmonary Capillary Wedge Pressure** Shown are the changes in sympathetic nervous activity assessed by norepinephrine spillover for the whole body (A and B), heart (C and D), and kidney (E and F), plotted against changes in left ventricular filling pressure assessed by pulmonary capillary wedge pressure. Graphs represent mean and 95% CI. Graphs A, C, and E show data for all participants. Graphs B, D, and F show data for the empagliflozin treatment group (in green) and the placebo treatment group (in red). Δ = change; NE = norepinephrine; PCWP = pulmonary capillary wedge pressure.

Figure 3 explores the relationship between change in SNA and change in PCWP using the composite pooled data for both groups. It should be noted that overall, a decrease in PCWP was correlated with an increase in whole-body NE spillover rate and cardiac SNA without change in renal NE spillover rate. An increase in PCWP was correlated with decreased whole-body NE spillover rate but oddly an increase in cardiac SNA and renal NE spillover rate.

**Central Illustration:**
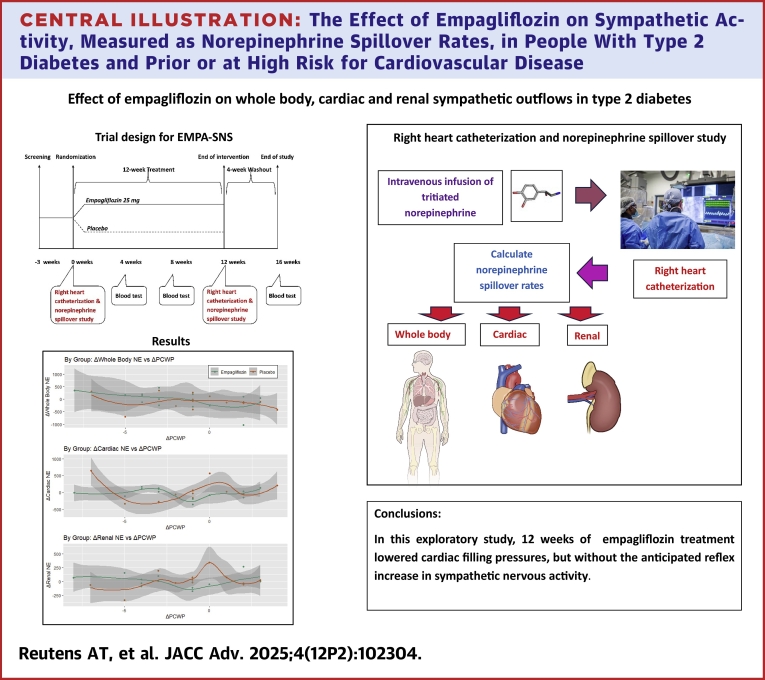
**The Effect of Empagliflozin on Sympathetic Activity, Measured as Norepinephrine Spillover Rates, in People With Type 2 Diabetes and Prior or at High Risk for Cardiovascular Disease** Empagliflozin, a sodium glucose co-transporter type 2 inhibitor, lowered cardiac filling pressures, but without the anticipated reflex increase in sympathetic nervous activity. Abbreviation as in Figure 1.

### Adverse events

Empagliflozin 25 mg daily for 12 weeks was well tolerated. The incidence of AEs was higher in the placebo arm compared to empagliflozin (Supplemental Table 2).

### Adherence

Participants’ compliance with investigational study product was excellent. In the empagliflozin arm, adherence assessed by pill count was 99.5%. In the placebo group, adherence was 97.4%.

## Discussion

This is the first double-blind, randomized, placebo-controlled study to assess the effect of SGLT2i on the SNS using the gold standard isotope dilution method to assess NE spillover kinetics, supported by relevant investigations including central hemodynamic assessment. The invasive nature of this technique is unique and provides insights not otherwise measurable. The main finding of this study was that empagliflozin treatment had no significant effect on SNS activity, while a small reduction in cardiac filling pressure was observed. Since such a filling pressure reduction would be anticipated to produce a reflex increase in SNA, empagliflozin in effect “uncoupled” this.

In this study, we studied whole body, cardiac, and renal sympathetic activity with tracer kinetic techniques using radiolabeled NE to determine the rate of appearance (spillover) of NE into the plasma compartment, with samples obtained at RHC in resting basal conditions. This method was used because quantitation of plasma NE alone (which has been used by other authors^35^, ^36^, ^37^) depends on both the rate of release of NE from the sympathetic nerve endings as well as the metabolic clearance rate, so may not directly reflect SNA.^38^

### Hemodynamic effects of empagliflozin

SGLT2is produce natriuresis, glycosuria, and osmotic diuresis. Accurate assessment of plasma volume using radioisotope dilution of ^131^I- or ^125^I-labeled human serum albumin was not done in our study. Other studies employing this technique in people with type 2 diabetes gave contradictory results of reduced plasma volume or no change. Empagliflozin reduced plasma volume by 138 mL in one study.^39^ Another study compared the effects on plasma volume of 12 weeks of dapagliflozin, hydrochlorothiazide, and placebo; dapagliflozin reduced plasma volume by ∼7% from baseline, but the other 2 treatments had no effect.^40^ However, the DAPASALT (DAPAgliflozin and SALT [sodium] excretion) study showed no significant effect of dapagliflozin on plasma volume.^41^

One possible mechanism by which SGLT2is may protect against heart failure is by reducing plasma volume without this activating the SNS. Evidence in favor of plasma volume reduction in the current study was provided by reductions in weight and PCWP, and increases in hematocrit and hemoglobin in the empagliflozin arm. However, the weight loss could have been partly due to fat loss,^42^, ^43^, ^44^, ^45^ and increased erythropoiesis, rather than hemoconcentration, may have caused the rise in hematocrit.^40^^,^^46^, ^47^, ^48^ There was a statistically significant but relatively small (1.78 mm Hg) decrease in PCWP measured at RHC in the empagliflozin-treated group compared to placebo in the current study even in the absence of symptomatic heart failure. PCWP moderately and directly correlated with total blood volume assessed by the indicator dilution method in a previous study.^49^ In our study population, which excluded people with severe heart failure, respiratory, or cardiac valvular disease, it would be reasonable to assume that reduced pulmonary arterial pressures reflected reduced intravascular volume.

Whether natriuresis and osmotic diuresis explain the beneficial effects of SGLT2i on cardiac health remain controversial,^50^ although it is known that modest reductions in intracardiac filling pressures do translate into improved HF outcomes.^51^ At the end of our study, the 24-hour urine volumes in those treated with empagliflozin were significantly higher than in those treated with placebo. There was no significant difference in natriuresis between the groups at the end of the study; however, we did not assess acute natriuresis. In other studies using SGLT2i, the diuretic effect was transient only, reflecting rapid renal compensation.^52^ In the DAPASALT study, there was no acute natriuresis observed in patients without heart failure who had normal renal function.^41^ Similarly, in patients with heart failure, Marton and colleagues recently found that dapagliflozin acutely increased glucose excretion without a parallel increase in sodium excretion.^53^ Stimulation of vasopressin release increased by 2 days and was still increased at 4 weeks after treatment was initiated. This increase in urine concentration caused a ∼10 mL/kg/day reduction in free water excretion. Furthermore, in a post hoc analysis of the EMPEROR-Preserved (Empagliflozin Outcome Trial in Patients With Chronic Heart Failure With Preserved Ejection Fraction) clinical trial, background diuretic use or dose did not affect improvements caused by empagliflozin on cardiovascular death or heart failure hospitalization, first and total heart failure hospitalizations, rate of decline in eGFR, and health status.^54^ Therefore, if SGLT2is function as diuretics, these effects may be weak or short-lived because of compensatory mechanisms.

### SNA did not increase with empagliflozin

Interestingly, we demonstrated that there was no change in global or regional (cardiac, renal) SNA after treatment with empagliflozin despite diuresis/natriuresis and reduced intracardiac filling pressures. Hyperinsulinemia per se in type 2 diabetes is causally related to increased SNS activity.^29^^,^^55^, ^56^, ^57^, ^58^ In our study, there was no significant alteration in blood pressure or heart rate with empagliflozin treatment. It was previously reported that SGLT2i administered to patients with acute myocardial infarction reduced blood pressure without causing an increase in heart rate,^17^^,^^59^ attributed to decreased cardiac SNA and increased parasympathetic activity as assessed by heart rate variability. Recent studies using MSNA gave differing results. After 12 weeks of dapagliflozin treatment, MSNA was reduced in people with type 2 diabetes with or without heart failure, but the reduction did not correlate with change in insulin resistance.^25^ Similar to our findings, there was no significant change in blood pressure in either heart failure or non-heart failure participants. By contrast, a study comparing 6 weeks of treatment with 25 mg/day empagliflozin or hydrochlorothiazide in people with type 2 diabetes but no heart failure^26^ found that systolic blood pressure decreased in both groups, but there was no change in resting MSNA. In a cross-over study, 14 days of treatment with 10 mg/day empagliflozin produced continued natriuresis and decreased plasma volume, without neurohormonal activation (albeit, measured by plasma NE). This was in contrast to what would be expected with conventional diuretics, which increase SNS activity.^60^ This is our key finding that despite lower filling pressure, there was no increase in SNS activity.

In our study, the lack of effect of empagliflozin on SNS activity may reflect its quite modest effect on contracting plasma volume (ie, it was not as strong a diuretic as previously anticipated, so that there was no lowering of blood pressure and then no reflex SNS activation). This would be in keeping with the results from Hallow and colleagues, who compared the effect of dapagliflozin with bumetanide on blood volume vs interstitial fluid volume.^61^ Dapagliflozin produced a 2-fold greater reduction in interstitial fluid volume compared to blood volume, whereas the loop diuretic caused only a 78% reduction of interstitial fluid volume compared to blood volume. This difference in effect, leading to less neurohormonal activation by SGLT2i, may be the reason for the effects of these agents in reducing heart failure incidence and improving cardiac outcomes.

### Study limitations

Our study has several limitations. First, because of the requirement for RHC, the sample size was modest and all participants came from one trial site. Recruitment was complicated by the COVID-19 epidemic, and prolonged, recurrent lockdowns, leading to slight underpowering for the primary endpoint. This limited the statistical power of this study, increasing the risk of type II error, particularly for the primary endpoint. Thus, a true effect of empagliflozin on SNS may have been missed. Second, the study population was mainly Caucasian, limiting the generalizability of the results to other ethnic groups. Third, the results may not be generalizable to women because most participants were men. Fourth, the study population had a relatively narrow range of HbA1c and body mass index, so the results may not be generalizable to people with higher metabolic risk. Fifth, we did not assess the acute effects (within 48 hours) on SNS activity. Sixth, the treatment period was relatively short at 12 weeks, which may not have been sufficient time for blood pressure effects to have fully developed. This effect may need ≥6 months.^62^ Seventh, the NE spillover rates were determined in supine, resting individuals. Clearly this does not reflect the situation of real life, when SNS is activated by standing up, mental and physical activity. Therefore, the effect on empagliflozin during normal living with its attendant heightened SNS activity may be different to our results. Eighth, we did not explicitly test each participant for autonomic dysautonomia. However, our study population was intended to replicate the participants in the EMPA-REG study^1^ which did not have checks for autonomic neuropathy. Moreover, severe neuropathy in our study population was unlikely given the absence of symptoms of postural hypotension or autonomic dysfunctional gastrointestinal symptoms on systems review. Therefore, we do not consider our study population to have been affected by any severe neuropathy. Sympathetic tone in autonomic failure is greatly reduced, as previously documented, below NE concentrations seen in our study.^63^

## Conclusions

In this study, 12 weeks of treatment with empagliflozin, when compared to placebo, did not change SNS activity measured with NE spillover kinetics, both for the whole body, as well as regionally for the heart and the kidney. These exploratory results provide insight into the mechanisms for cardiovascular protection provided by SGLT2i because although diuresis persisted, the SNS activity did not increase.

## Funding support and author disclosures

This trial was supported by an unrestricted investigator-initiated study grant from 10.13039/100001003Boehringer Ingelheim Pty. Ltd (78 Waterloo Road, North Ryde, NSW 2113, Australia). The study sponsor was not involved in designing the study, data collection, analysis, or report writing. The blinded investigational medicinal product was also supplied by 10.13039/100001003Boehringer Ingelheim. The 10.13039/501100000925National Health and Medical Research Council of Australia supports Professor Shaw with a Leadership Investigator Grant and Professor Kaye with a Principal Senior Fellowship. The Baker Heart and Diabetes Institute is supported in part by the Victorian Government's Operational Infrastructure Support Program. The authors have reported that they have no relationships relevant to the contents of this paper to disclose.

## References

[1] Zinman B., Wanner C., Lachin J.M. (2015). Empagliflozin, cardiovascular outcomes, and mortality in type 2 diabetes. N Engl J Med.

[2] Neal B., Perkovic V., Mahaffey K.W. (2017). Canagliflozin and cardiovascular and renal events in type 2 diabetes. N Engl J Med.

[3] Wiviott S.D., Raz I., Bonaca M.P. (2019). Dapagliflozin and cardiovascular outcomes in type 2 diabetes. N Engl J Med.

[4] Usman M.S., Siddiqi T.J., Anker S.D. (2023). Effect of sglt2 inhibitors on cardiovascular outcomes across various patient populations. J Am Coll Cardiol.

[5] McMurray J.J.V., Solomon S.D., Inzucchi S.E. (2019). Dapagliflozin in patients with heart failure and reduced ejection fraction. N Engl J Med.

[6] Packer M., Anker S.D., Butler J. (2020). Cardiovascular and renal outcomes with empagliflozin in heart failure. N Engl J Med.

[7] Packer M., Butler J., Zannad F. (2021). Effect of empagliflozin on worsening heart failure events in patients with heart failure and preserved ejection fraction: emperor- preserved trial. Circulation.

[8] Solomon S.D., McMurray J.J.V., Claggett B. (2022). Dapagliflozin in heart failure with mildly reduced or preserved ejection fraction. N Engl J Med.

[9] Nuffield Department of Population Health Renal Studies Group: SGLT2 Inhibitor Meta-Analysis Cardio-Renal Trialists' Consortium (2022). Impact of diabetes on the effects of sodium glucose co-transporter-2 inhibitors on kidney outcomes: collaborative meta-analysis of large placebo-controlled trials. Lancet.

[10] Inzucchi S.E., Zinman B., Fitchett D. (2018). How does empagliflozin reduce cardiovascular mortality? Insights from a mediation analysis of the empa-reg outcome trial. Diabetes Care.

[11] Rodriguez-Valadez J.M., Tahsin M., Masharani U. (2024). Potential mediators for treatment effects of novel diabetes medications on cardiovascular and renal outcomes: a meta-regression analysis. J Am Heart Assoc.

[12] Bechmann L.E., Emanuelsson F., Nordestgaard B.G., Benn M. (2023). Genetic variation in solute carrier family 5 member 2 mimicking sodium-glucose co-transporter 2- inhibition and risk of cardiovascular disease and all-cause mortality: reduced risk not explained by lower plasma glucose. Cardiovasc Res.

[13] Mudaliar S., Alloju S., Henry R.R. (2016). Can a shift in fuel energetics explain the beneficial cardiorenal outcomes in the empa-reg outcome study? A unifying hypothesis. Diabetes Care.

[14] Buttice L., Ghani M., Suthakar J. (2024). The effect of sodium-glucose cotransporter-2 inhibitors on inflammatory biomarkers: a meta-analysis of randomized controlled trials. Diabetes Obes Metab.

[15] Requena-Ibáñez J.A., Santos-Gallego C.G., Rodriguez-Cordero A. (2021). Mechanistic insights of empagliflozin in nondiabetic patients with hfref: from the empa-tropism study. JACC Heart Fail.

[16] Sano M. (2018). A new class of drugs for heart failure: Sglt2 inhibitors reduce sympathetic overactivity. J Cardiol.

[17] Scheen A.J. (2019). Effect of sglt2 inhibitors on the sympathetic nervous system and blood pressure. Curr Cardiol Rep.

[18] Packer M. (1992). The neurohormonal hypothesis: a theory to explain the mechanism of disease progression in heart failure. J Am Coll Cardiol.

[19] Hartupee J., Mann D.L. (2017). Neurohormonal activation in heart failure with reduced ejection fraction. Nat Rev Cardiol.

[20] (1999). The cardiac insufficiency bisoprolol study ii (Cibis-ii): a randomised trial. Lancet.

[21] Packer M., Fowler M.B., Roecker E.B. (2002). Effect of carvedilol on the morbidity of patients with severe chronic heart failure: results of the carvedilol prospective randomized cumulative survival (COPERNICUS) study. Circulation.

[22] Kim H.K., Ishizawa R., Fukazawa A. (2022). Dapagliflozin attenuates sympathetic and pressor responses to stress in young prehypertensive spontaneously hypertensive rats. Hypertension.

[23] Gueguen C., Burke S.L., Barzel B. (2020). Empagliflozin modulates renal sympathetic and heart rate baroreflexes in a rabbit model of diabetes. Diabetologia.

[24] Jordan J., Tank J., Heusser K. (2017). The effect of empagliflozin on muscle sympathetic nerve activity in patients with type ii diabetes mellitus. J Am Soc Hypertens.

[25] Hamaoka T., Murai H., Hirai T. (2021). Different responses of muscle sympathetic nerve activity to dapagliflozin between patients with type 2 diabetes with and without heart failure. J Am Heart Assoc.

[26] Heusser K., Tank J., Diedrich A., Fischer A., Heise T., Jordan J. (2023). Randomized trial comparing sglt2 inhibition and hydrochlorothiazide on sympathetic traffic in type 2 diabetes. Kidney Int Rep.

[27] Esler M., Blombery P., Leonard P., Jennings G., Korner P. (1982). Radiotracer methodology for the simultaneous estimation of total, and renal, sympathetic nervous system activity in humans. Clin Sci.

[28] Esler M., Jackman G., Leonard P. (1980). Determination of noradrenaline uptake, spillover to plasma and plasma concentration in patients with essential hypertension. Clin Sci.

[29] Straznicky N.E., Grima M.T., Sari C.I. (2012). Neuroadrenergic dysfunction along the diabetes continuum: a comparative study in obese metabolic syndrome subjects. Diabetes.

[30] Esler M., Lambert G., Brunner-La Rocca H.P., Vaddadi G., Kaye D. (2003). Sympathetic nerve activity and neurotransmitter release in humans: translation from pathophysiology into clinical practice. Acta Physiol Scand.

[31] Esler M. (1993). Clinical application of noradrenaline spillover methodology: delineation of regional human sympathetic nervous responses. Pharmacol Toxicol.

[32] Esler M., Jennings G., Korner P., Blombery P., Sacharias N., Leonard P. (1984). Measurement of total and organ-specific norepinephrine kinetics in humans. Am J Physiol.

[33] Esler M. (1982). Assessment of sympathetic nervous function in humans from noradrenaline plasma kinetics. Clin Sci.

[34] Friberg P., Meredith I., Jennings G., Lambert G., Fazio V., Esler M. (1990). Evidence for increased renal norepinephrine overflow during sodium restriction in humans. Hypertension.

[35] Daniele G., Solis-Herrera C., Dardano A. (2020). Increase in endogenous glucose production with sglt2 inhibition is attenuated in individuals who underwent kidney transplantation and bilateral native nephrectomy. Diabetologia.

[36] Cherney D.Z., Perkins B.A., Soleymanlou N. (2014). The effect of empagliflozin on arterial stiffness and heart rate variability in subjects with uncomplicated type 1 diabetes mellitus. Cardiovasc Diabetol.

[37] Herring R.A., Parsons I., Shojaee-Moradie F. (2023). Effect of dapagliflozin on cardiac function and metabolic and hormonal responses to exercise. J Clin Endocrinol Metab.

[38] Esler M., Jackman G., Bobik A. (1979). Determination of norepinephrine apparent release rate and clearance in humans. Life Sci.

[39] Griffin M., Rao V.S., Ivey-Miranda J. (2020). Empagliflozin in heart failure: diuretic and cardiorenal effects. Circulation.

[40] Lambers Heerspink H.J., de Zeeuw D., Wie L., Leslie B., List J. (2013). Dapagliflozin a glucose- regulating drug with diuretic properties in subjects with type 2 diabetes. Diabetes Obes Metab.

[41] Scholtes R.A., Muskiet M.H.A., van Baar M.J.B. (2021). Natriuretic effect of two weeks of dapagliflozin treatment in patients with type 2 diabetes and preserved kidney function during standardized sodium intake: results of the dapasalt trial. Diabetes Care.

[42] Abdelgani S., Khattab A., Adams J. (2024). Empagliflozin reduces liver fat in individuals with and without diabetes. Diabetes Care.

[43] Veelen A., Andriessen C., Op den Kamp Y. (2023). Effects of the sodium-glucose cotransporter 2 inhibitor dapagliflozin on substrate metabolism in prediabetic insulin resistant individuals: a randoSzed, double-blind crossover trial. Metabolism.

[44] Breder I., Wolf V.L.W., Soares A.A.S. (2022). Dapagliflozin reduces adiposity and increases adiponectin in patients with type 2 diabetes and atherosclerotic disease at short-term: an active-controlled randomised trial. Diabetes Metab.

[45] Bolinder J., Ljunggren Ö., Kullberg J. (2012). Effects of dapagliflozin on body weight, total fat mass, and regional adipose tissue distribution in patients with type 2 diabetes mellitus with inadequate glycemic control on metformin. J Clin Endocrinol Metab.

[46] Fuchs Andersen C., Omar M., Glenthøj A. (2023). Effects of empagliflozin on erythropoiesis in heart failure: data from the empire hf trial. Eur J Heart Fail.

[47] Mazer C.D., Hare G.M.T., Connelly P.W. (2020). Effect of empagliflozin on erythropoietin levels, iron stores, and red blood cell morphology in patients with type 2 diabetes mellitus and coronary artery disease. Circulation.

[48] Yamada T., Sakaguchi K., Okada Y. (2021). Analysis of time-dependent alterations of parameters related to erythrocytes after ipragliflozin initiation. Diabetol Int.

[49] Miller W.L., Sorimachi H., Grill D.E., Fischer K., Borlaug B.A. (2021). Contributions of cardiac dysfunction and volume status to central haemodynamics in chronic heart failure. Eur J Heart Fail.

[50] Packer M. (2024). Sglt2 inhibition: neither a diuretic nor a natriuretic. J Am Coll Cardiol.

[51] Zile M.R., Bennett T.D., El Hajj S. (2017). Intracardiac pressures measured using an implantable hemodynamic monitor. Circ Heart Fail.

[52] Packer M., Wilcox C.S., Testani J.M. (2023). Critical analysis of the effects of sglt2 inhibitors on renal tubular sodium, water and chloride homeostasis and their role in influencing heart failure outcomes. Circulation.

[53] Marton A., Saffari S.E., Rauh M. (2024). Water conservation overrides osmotic diuresis during sglt2 inhibition in patients with heart failure. J Am Coll Cardiol.

[54] Butler J., Usman M.S., Filippatos G. (2023). Safety and efficacy of empagliflozin and diuretic use in patients with heart failure and preserved ejection fraction: a post hoc analysis of the emperor-preserved trial. JAMA Cardiol.

[55] Huggett R.J., Scott E.M., Gilbey S.G., Stoker J.B., Mackintosh A.F., Mary D.A.S.G. (2003). Impact of type 2 diabetes mellitus on sympathetic neural mechanisms in hypertension. Circulation.

[56] Straznicky N.E., Lambert E.A., Grima M.T. (2014). The effects of dietary weight loss on indices of norepinephrine turnover: modulatory influence of hyperinsulinemia. Obesity.

[57] Straznicky N.E., Grima M.T., Sari C.I. (2016). Comparable attenuation of sympathetic nervous system activity in obese subjects with normal glucose tolerance, impaired glucose tolerance, and treatment naïve type 2 diabetes following equivalent weight loss. Front Physiol.

[58] Rahmouni K., Haynes W.G., Morgan D.A., Mark A.L. (2003). Role of melanocortin-4 receptors in mediating renal sympathoactivation to leptin and insulin. J Neurosci.

[59] Kubota Y., Yamamoto T., Tara S. (2018). Effect of empagliflozin versus placebo on cardiac sympathetic activity in acute myocardial infarction patients with type 2 diabetes mellitus: rationale. Diabetes Ther.

[60] Francis G.S., Siegel R.M., Goldsmith S.R., Olivari M.T., Levine T.B., Cohn J.N. (1985). Acute vasoconstrictor response to intravenous furosemide in patients with chronic congestive heart failure. Activation of the neurohumoral axis. Ann Intern Med.

[61] Hallow K.M., Helmlinger G., Greasley P.J., McMurray J.J.V., Boulton D.W. (2018). Why do sglt2 inhibitors reduce heart failure hospitalization? A differential volume regulation hypothesis. Diabetes Obes Metab.

[62] Ferdinand K.C., Izzo J.L., Lee J. (2019). Antihyperglycemic and blood pressure effects of empagliflozin in black patients with type 2 diabetes mellitus and hypertension. Circulation.

[63] Meredith I.T., Eisenhofer G., Lambert G.W., Jennings G.L., Thompson J., Esler M.D. (1992). Plasma norepinephrine responses to head-up tilt are misleading in autonomic failure. Hypertension.

