# Supplementary material for: Effect of Empagliflozin on Whole Body, Cardiac, and Renal Sympathetic Outflows in Type 2 Diabetes
Source: JACC Adv. 2025 Nov 8;4(12):102304. doi: 10.1016/j.jacadv.2025.102304 (PMC12639828; doi:10.1016/j.jacadv.2025.102304)
Supplement: Supplemental Material [file mmc1.pdf]

**Participant Eligibility Criteria In The EMPA-SNS Trial**

**<u>Participant Inclusion Criteria</u>**

1) Capable of understanding the content of and able voluntarily to provide a personally signed and dated written informed consent form.

2) Stated willingness to comply with all study procedures and availability for the duration of the study.

3) Male or female, aged 18-85 years inclusive.

4) Diagnosed with type 2 diabetes.

5) HbA1c between 6.5% (48 mmol/mol) and 10.0% (86 mmol/mol) inclusive, at Visit 1.

6) Antidiabetic therapy has to be unchanged for 6 weeks before Visit 1.

a) If insulin is part of the background therapy, this means no change in the dose of insulin in excess of ±10% of the average daily dose.

b) For oral antidiabetic medications, this means no change in the number or class of glucose-lowering drugs, and no change in excess of ±20% of the average daily dose.

c) For GLP-1 receptor agonists, this means no change in dose of the average daily dose.

7) eGFR ≥ 30 mL/min/1.73 m2, as calculated by the CKD-EPI creat formula, at Visit 1.

8) Body mass index between 20-40 kg/m2 inclusive, at Visit 1.

9) In addition to the above described criteria, participants must have high cardiovascular risk, defined as at least one of the following: A] confirmed history of coronary artery disease, or B] age ≥ 30 years with documented symptomatic atherosclerotic non-coronary cardiovascular events, or C] age ≥ 50 years with 2 or more defined risk factors identified at the screening visit.

A] Confirmed history of coronary artery disease is defined as at least one of the following:

a) Confirmed history of myocardial infarction (>2 months prior to Visit 1).

b) Evidence of multivessel coronary artery disease, in 2 or more major coronary arteries, irrespective of the revascularisation status, i.e.

i) Either the presence of a significant stenosis (imaging evidence of at least 50% narrowing of the luminal diameter measured during a coronary angiography or a multi-sliced computed tomography angiography), in 2 or more major coronary arteries,

or

ii) a previous revascularisation (percutaneous transluminal coronary angioplasty with or without stent, or coronary artery bypass grafting) at least 2 months ago, in 2 or more major coronary arteries,

or

iii) the combination of previous revascularisation in one major coronary artery at least 2 months ago (percutaneous transluminal coronary angioplasty with or without stent, or coronary artery bypass grafting), and the presence of a significant stenosis in another major coronary artery (imaging evidence of at least 50% narrowing of the luminal diameter measured during a coronary angiography or a multi-sliced computed tomography angiography),

Note: A disease affecting the left main coronary artery is considered as a 2-vessel disease.

c) Evidence of a single vessel coronary artery disease with:

i) The presence of a significant stenosis i.e. the imaging evidence of at least 50% narrowing of the luminal diameter of one major coronary artery in participants not subsequently successfully revascularised (measured during a coronary angiography or a multi-sliced computed tomography angiography)

ii) And at least one of the following (either (1) or (2) below):

(1) A positive non-invasive stress test, confirmed by either:

- A positive exercise tolerance test in participants without a complete left bundle

branch block, Wolff-Parkinson-White syndrome, or paced ventricular rhythm,

or

• A positive stress echocardiography showing regional systolic wall motion abnormalities,

or

• A positive scintigraphic test showing stress-induced ischaemia, i.e. the development of transient perfusion defects during myocardial perfusion imaging;

(2) Participant discharged from hospital with a documented diagnosis of unstable angina within 12 months prior to Visit 1.

d) Last episode of unstable angina >2 months prior to Visit 1, with confirmed evidence of coronary multivessel or single vessel disease as defined above.

B] Age ≥ 30 years with documented symptomatic atherosclerotic non-coronary cardiovascular events, defined as at least one of the following: stroke, peripheral revascularisation (angioplasty or surgery); symptomatic with documented haemodynamically-significant carotid or peripheral vascular disease.

C] Age ≥ 50 years with 2 or more of the following risk factors at Visit 1: duration of type 2 diabetes ≥ 10 years; participant is on at least one anti-hypertensive medication prescribed by a doctor for blood-pressure lowering; the average of 3 readings of systolic blood pressure at Visit 1 is > 140 mm Hg; documented albuminuria within 12 months of Visit 1 (defined as at least one urine albumin to creatinine ratio ≥ 3 mg/mmol in the last 12 months, with at least one other abnormal albumin to creatinine ratio ≥ 3 mg/mmol documented in the history); documented HDL-cholesterol < 1.0 mmol/L within 12 months of Visit 1; low-density lipoprotein cholesterol (LDL-Cl) >3.36 mmol/L) within last 12 months (verified by

documentation of laboratory value LDL-C > 3.36 mmol/L); on lipid-lowering therapy prescribed by a doctor.

10) Willing to practice highly effective methods of birth control (both males who have partners of childbearing potential and females of childbearing potential) during the screening period, while taking investigational product and for at least 90 days after the last dose of investigational product is ingested. Women of childbearing potential are female participants who are not surgically sterile (no history of bilateral tubal ligation, hysterectomy, or bilateral salpingo-oophorectomy), and are not postmenopausal for at least 1 year. Furthermore, male study participants must also not donate sperm from day of randomisation until 90 days after the last dose of investigational product.

11) The only permitted antidepressant medications are serotonin and norepinephrine reuptake inhibitors (SNRIs) or selective serotonin reuptake inhibitors (SSRIs).

a) If on antidepressant medication SSRI or SNRI as part of background therapy, the dose has to be unchanged for 12 weeks before Visit 1.

**Participant Exclusion Criteria**

1) History of type 1 diabetes.

2) Uncontrolled hyperglycaemia with fasting plasma glucose level > 13.3 mmol/L during screening and confirmed by a second measurement performed on a separate day.

3) History of 1 or more episodes of ketoacidosis or hyperosmolar state/coma requiring hospitalisation within the 6 months prior to Visit 1.

4) Ongoing therapy with an SGLT2 inhibitor or pioglitazone.

5) Previously intolerant of an SGLT2 inhibitor.

6) Acute coronary syndrome, stroke or TIA within 2 months prior to Visit 1.

7) On monoamine oxidase inhibitors or tricyclic antidepressants.

8) Diagnosed hypertrophic obstructive cardiomyopathy, dilated cardiomyopathy or restrictive cardiomyopathy.

9) New York Heart Association class III or IV heart failure.

10) Valvular heart disease, moderate or severe, i.e. Stage B moderate or severe, Stage C or Stage D, as defined by the current American Heart Association clinical guidelines9.

11) Current smoker.

12) Indication of liver disease, defined by serum levels of either ALT, AST, or alkaline phosphatase above 3 x upper limit of normal (ULN) at Visit 1 or bilirubin above 1.5 x the ULN measured at Visit 1.

13) Planned cardiac surgery or angioplasty within 19 weeks of Visit 1

14) Bariatric surgery within the past two years, or history of other gastrointestinal surgeries that induce chronic malabsorption.

15) Treatment with anti-obesity drugs 3 months prior to Visit 1 (e.g. orlistat, zonisamide, topiramate, phentermine, lorcaserin, bupropion, naltrexone, either alone or in combination for the purpose of weight loss) or any other treatment at the time of screening (e.g. aggressive diet regimen, etc.) leading to unstable body weight. Unstable body weight is defined as more than 5 kg self-reported change within the 3 months before Visit 1.

16) Have any haematological condition that may interfere with HbA1c measurement (e.g. haemolytic anaemias, haemoglobinopathy).

17) History of an active or untreated malignancy, or in remission from a clinically significant malignancy (other than basal or squamous cell skin cancer, in situ carcinoma of the cervix, or in situ prostate cancer) for less than 5 years prior to Visit 1, or are receiving or planning to receive therapy for cancer, at Visit 1.

18) Are receiving chronic (>2 weeks or 14 days) systemic glucocorticoid therapy (excluding topical, intra-ocular, intranasal, or inhaled preparations) or have received such therapy within 4 weeks of Visit 1.

19) Change in dosage of thyroid replacement hormone within 6 weeks prior to Visit 1.

20) Other endocrine disorder, with the exception of type 2 diabetes and hypothyroidism on stable thyroid replacement dose.

21) Pre-menopausal women (last menstruation ≤ 1 year prior to informed consent) who:

a) are nursing or pregnant or

b) are of child-bearing potential and are not practicing an acceptable method of birth control, or do not plan to continue using this method throughout the study and for 30 days after end of treatment, and do not agree to submit to periodic pregnancy testing during participation in the trial.

22) Inadequately controlled arterial blood pressure, defined as SBP >160 mmHg at Visit 1 or DBP > 100 mm Hg at Visit 1.

23) Current genito-urinal infection or history of genito-urinal infection within 2 weeks prior to Visit 1 (but not simple asymptomatic bacteriuria).

24) The occurrence of any acute infection requiring systemic antibiotic therapy within the 2 weeks prior to Visit 1, or known infection with hepatitis B, hepatitis C, or human immunodeficiency virus (HIV) infection.

25) Patients with any rare hereditary condition of galactose intolerance, e.g. galactosaemia.

26) Alcohol or drug abuse within the 3 months prior to Visit 1 that would interfere with trial participation.

27) Administration of any investigational product within 30 days or within 5 half-lives of the investigational agent (whichever is longer) of Visit 1, or currently participating in another trial (involving an investigational drug and/or follow-up).

28) Any condition which, in the opinion of the Investigator, constitutes a risk or contraindication for the participation of the participant in the study, or that could interfere with the study objectives, conduct, or evaluation. This includes participants unlikely to comply with the study protocol (e.g. an inability and unwillingness to participate in adequate training, an uncooperative attitude, inability to return for follow-up visits, or unlikelihood of completing the study).

29) Persons employed by the Sponsor, Boehringer Ingelheim.

30) Persons who are Investigator site personnel directly affiliated with this study and/or their immediate families. Immediate family is defined as a spouse, parent, child, or sibling, whether biological or adopted.

31) Participants who, in the opinion of the investigator and based on the participant's comorbid profile, are likely to have a change in dose of GLP-1 receptor agonist during the study.

## Supplemental Table 1.

## Hematology results

| Parameter | Empagliflozin | | Placebo | | Estimated treatment difference at 12 weeks adjusted for baseline, empagliflozin vs. placebo, | | p-value |
|---|---|---|---|---|---|---|---|
| | Baseline Mean (SD) | 12 weeks Mean (SD) | Baseline Mean (SD) | 12 weeks Mean (SD) | Estimate (95% CI) | | |
| Hb | 138.2 (12.80) | 138.73 (13.01) | 137.12 (7.97) | 135.12 (8.26) | 2.461 | (-2.082, 7.004,) | 0.298 |
| WBC | 7.35 (2.46) | 7.00 (2.07) | 7.27 (1.77) | 7.12 (1.72) | -0.256 | (-1.256, 0.743) | 0.619 |
| Platelets | 246.80 (101.58) | 276.53(141.09) | 250.71 (58.20) | 260.88 (58.27) | 16.914 | (-12.096,45.924) | 0.264 |
| Hct | 0.41 (0.03) | 0.42 (0.03) | 0.41 (0.02) | 0.40 (0.02) | 0.019 | (0.004,0.033) | **0.018** |
| MCV | 89.53 (6.21) | 89.20 (7.05) | 91.29 (5.43) | 91.00 (5.66) | 0.392 | (-0.911,1.696) | 0.561 |
| MCH | 30.08 (2.83) | 29.31 (2.95) | 30.51 (2.15) | 30.63 (2.24) | -0.894 | (-1.439, -0.349) | **0.003** |
| MCHC | 334.67 (11.93) | 327.73 (8.46) | 335.06 (9.14) | 336.75 (9.16) | -9.123 | (-13.791,-4.455) | **0.001** |
| RBC | 4.63 (0.52) | 4.77 (0.60) | 4.50 (0.41) | 4.42 (0.45) | 0.193 | (0.002,0.384) | 0.059 |
| RDW | 13.72 (1.14) | 13.99 (1.31) | 13.51 (0.99) | 13.34 (0.85) | 0.383 | (0.044,0.721) | **0.035** |
| Neutrophils | 4.67 (2.25) | 4.59 (1.80) | 4.37 (1.44) | 4.14 (1.21) | 0.132 | (-0.517,0.78) | 0.694 |
| Monocytes | 0.62 (0.22) | 0.59 (0.15) | 0.59 (0.18) | 0.55 (0.19) | 0.015 | (-0.061,0.092) | 0.698 |
| Lymphocytes | 1.78 (0.56) | 1.56 (0.45) | 2.05 (0.47) | 2.13 (0.53) | -0.406 | (-0.693, -0.118) | **0.010** |
| Eosinophils | 0.24 (0.14) | 0.22 (0.10) | 0.21 (0.20) | 0.22 (0.12) | -0.006 | (-0.06,0.047) | 0.817 |
| Basophils | 0.04 (0.01) | 0.05 (0.02) | 0.05 (0.02) | 0.05 (0.02) | 0.004 | (-0.007,0.015) | 0.523 |

SD indicates standard deviation; CI indicates confidence interval, Hb hemoglobin, WBC white blood cell count, Hct hematocrit, MCV mean corpuscular volume, MCH mean corpuscular volume, MCHC mean corpuscular hemoglobin concentration, RBC red blood cell count, RDW red cell distribution width

## Supplemental Table 2.

**Listing of adverse events**

| | | Number of participants with at least one Adverse Event | | | | | |
|---|---|---|---|---|---|---|---|
| **System Organ Class** | **Preferred Term** | **Empagliflozin** | | | **Placebo** | | |
| | | | **Severity** | **Related** | | **Severity** | **Related** |
| **Cardiac disorders** | Palpitations | | | | 1 | Moderate | No |
| **Eye disorders** | Conjunctivitis | | | | 1 | Mild | No |
| | Diabetic retinopathy | | | | 1 | Moderate | No |
| | Vitreous floaters | 1 | Mild | No | | | |
| **Gastrointestinal disorders** | Constipation | 1 | Mild | No | | | |
| **Infections and infestations** | Cellulitis | 1 | Severe and SAE | No | | | |
| | Common cold | | | | 2 | Mild | No |
| | Folliculitis | | | | 1 | Mild | No |
| | Pharyngitis | | | | 1 | Mild | No |
| | Shingles | | | | 1 | Moderate | No |
| | Upper respiratory tract infection | | | | 1 | Mild | No |

| System Organ Class | Preferred Term | Number of participants with at least one Adverse Event | | | | | |
|---|---|---|---|---|---|---|---|
| | | Empagliflozin | | | Placebo | | |
| | | | Severity | Related | | Severity | Related |
| **Injury, poisoning and procedural complications** | Adhesive tape allergy | | | | 1 | Mild | Yes* |
| | Bruise | | | | 1 | Moderate | No |
| | Dog bite caused fracture of toe | | | | 1 | Severe and SAE | No |
| | Pain in arm | | | | 1 | Mild | Yes* |
| | Swelling in arm | | | | 1 | Moderate | Possibly* |
| | Syncope | 1 | Moderate | Yes* | | | |
| **Musculoskeletal and connective tissue disorders** | Intervertebral disc herniation | | | | 1 | Severe | No |
| **Nervous system disorders** | Dizziness | 1 | Mild | No | | | |
| **Renal and urinary disorders** | Polyuria | 1 | Mild | Yes | | | |
| **Skin and subcutaneous tissue disorders** | Rash | | | | 1 | Mild | Yes |
| | Redness of face | 1 | Mild | No | | | |

*** indicates related to study procedure**

| Patient | Study | ARTERIAL Catechols | | TOTAL NORADRENALINE PLASMA KINETICS | | | | | RENAL ISOTOPE-DERIVED NORADRENALINE KINETICS | | | | | | | CARDIAC ISOTOPE-DERIVED NORADRENALINE KINETICS | | | | |
|---|---|---|---|---|---|---|---|---|---|---|---|---|---|---|---|---|---|---|---|---|
| No. | Day | Noradr. pg/ml | Adren. pg/ml | Trac. Inf. dpm/min | ART 3HN dpm/ml | NA Clearance L/min | **NA Spillover ng/min** | | ART NA pg/ml | Renal vein NA pg/ml | 3H NA EXTRACT % | Renal Plasma Flow ml/min | **Renal NA Spillover ng/min** | | | ART NA pg/ml | Coronary Sinus NA pg/ml | 3H NA EXTRACT % | **3H Extraction Corrected NA Grad. pg/ml** | COMMENTS ........... |
| 001- | Baseline | 461 | 124 | 1.63x10$^{5}$ | 72 | 2.29 | **1056** | | 613 | 487 | 0.79 | 635 | **228** | | | 411 | 599 | 0.34 | **320** | |
| | Endpoint | 497 | 80 | 1.31x10$^{5}$ | 46 | 2.89 | **1411** | | 493 | 528 | 0.79 | 703 | **298** | | | 460 | 692 | 0.18 | **315** | |
| 002- | Baseline | 409 | 88 | 1.44x10$^{5}$ | 71 | 2.14 | **875** | | 413 | 324 | 0.8 | 662 | **160** | | | 394 | 658 | 0.31 | **382** | |
| | Endpoint | 524 | 134 | 1.52x10$^{5}$ | 77 | 1.98 | **1038** | | 553 | 545 | 0.68 | 861 | **317** | | | 495 | 667 | 0.19 | **266** | |
| 003- | Baseline | 84 | 82 | 2.25x10$^{5}$ | 118 | 1.9 | **160** | | 83 | 82 | 0.28 | 716 | **16** | | | 84 | 70 | 0.83 | **56** | **Vasovagal syndrome ***** |
| | Endpoint | 256 | 82 | 1.40x10$^{5}$ | 73 | 1.91 | **489** | | 219 | 267 | 0.49 | 1021 | **114** | | | 292 | 152 | 0.51 | **9** | |
| 004- | Baseline | 451 | 142 | 1.72x10$^{5}$ | 93 | 1.85 | **834** | | NOT | RENAL | VEIN | ....... | **.......** | ....... | ....... | 492 | 337 | 0.5 | **91** | **Not Renal Vein ***** |
| | Endpoint | 251 | 133 | 1.55x10$^{5}$ | 97 | 1.6 | **402** | | 235 | 351 | 0.38 | 744 | **153** | | | 266 | 435 | 0.5 | **302** | |
| 006- | Baseline | 309 | 111 | 1.54x10$^{5}$ | 72 | 2.14 | **661** | | 293 | 386 | 0.34 | 1051 | **202** | | | 325 | 286 | 0.53 | **131** | |
| | Endpoint | 357 | 87 | 4.47x10$^{5}$ | 210 | 2.13 | **760** | | 319 | 575 | 0.34 | 1293 | **471** | | | 394 | 351 | 0.53 | **166** | |
| 007- | Baseline | 894 | 95 | 5.91x10$^{5}$ | 287 | 2.06 | **1842** | | 798 | 1150 | 0.31 | 693 | **415** | | | 990 | 1324 | 0.5 | **829** | |
| | Endpoint | 1175 | 70 | 3.76x10$^{5}$ | 226 | 1.67 | **1962** | | 1192 | 1897 | 0.28 | 726 | **754** | | | 1157 | 1555 | 0.86 | **1393** | |
| 008- | Baseline | 469 | 204 | 5.20x10$^{5}$ | 263 | 1.98 | **929** | | 485 | 588 | 0.61 | 721 | **288** | | | 453 | 675 | 0.44 | **421** | |
| | Endpoint | 536 | 120 | 5.48x10$^{5}$ | 238 | 2.3 | **1232** | | 559 | 773 | 0.23 | 654 | **224** | | | 512 | 1135 | 0.44 | **1073** | |
| 010- | Baseline | 379 | 124 | 3.69x10$^{5}$ | 155 | 2.34 | **887** | | 349 | 422 | 0.31 | 474 | **86** | | | 409 | 544 | 0.56 | **364** | |
| | Endpoint | 292 | 70 | 5.10x10$^{5}$ | 314 | 1.62 | **473** | | 292 | 445 | 0.31 | 314 | **76** | | | 291 | 417 | 0.25 | **199** | |
| 011- | Baseline | 570 | 89 | 4.29x10$^{5}$ | 144 | 2.98 | **1699** | | 501 | 710 | 0.37 | 680 | **268** | | | 639 | 904 | 0.82 | **789** | |
| | Endpoint | 417 | 70 | 2.11x10$^{5}$ | 133 | 1.59 | **663** | | 384 | 754 | 0.3 | 492 | **239** | | | 451 | 718 | 0.82 | **637** | |
| 012- | Baseline | 510 | 139 | 3.90x10$^{5}$ | 174 | 2.24 | **1142** | | 543 | 828 | 0.8 | 820 | **590** | | | 476 | 727 | 1 | **727** | |
| | Endpoint | 334 | 107 | 3.31x10$^{5}$ | 253 | 1.31 | **438** | | 275 | 371 | 0.8 | 806 | **255** | | | 394 | 394 | 1 | **394** | |
| 015- | Baseline | 448 | 87 | 9.04x10$^{5}$ | 424 | 2.13 | **954** | | 414 | 774 | 0.29 | 600 | **288** | | | 481 | 755 | 0.54 | **534** | |
| | Endpoint | 415 | 95 | 4.73x10$^{5}$ | 170 | 2.78 | **1154** | | 435 | 598 | 0.28 | 412 | **112** | | | 394 | 507 | 0.59 | **345** | |
| 016- | Baseline | 380 | 44 | 8.16x10$^{5}$ | 370 | 2.21 | **840** | | 378 | 492 | 0.29 | 528 | **118** | | | 382 | 722 | 0.14 | **393** | |
| | Endpoint | 466 | 53 | 8.82x10$^{5}$ | 573 | 1.54 | **718** | | 480 | 527 | 0.43 | 614 | **156** | | | 451 | 772 | 0.53 | **560** | |
| 017- | Baseline | 269 | 50 | 7.95x10$^{5}$ | 318 | 2.5 | **673** | | 247 | 289 | 0.37 | 755 | **101** | | | 290 | 432 | 0.63 | **325** | |
| | Endpoint | 238 | 57 | 6.74x10$^{5}$ | 313 | 2.15 | **512** | | 202 | 192 | 0.44 | 602 | **47** | | | 274 | 423 | 0.49 | **283** | |
| 018- | Baseline | 464 | 36 | 7.45x10$^{5}$ | 365 | 2.04 | **947** | | 399 | 640 | 0.63 | 550 | **271** | | | 529 | 553 | 0.88 | **490** | |
| | Endpoint | 300 | 44 | 7.86x10$^{5}$ | 365 | 2.15 | **645** | | 313 | 395 | 0.63 | 578 | **161** | | | 288 | 173 | 0.85 | **130** | |
| 021- | Baseline | 391 | 44 | 6.48x10$^{5}$ | 389 | 1.67 | **653** | | 344 | 465 | 0.26 | 579 | **122** | | | 438 | 530 | 0.46 | **293** | |
| | Endpoint | 250 | 32 | 7.13x10$^{5}$ | 442 | 1.61 | **403** | | 238 | 329 | 0.56 | 574 | **129** | | | 261 | 398 | 0.69 | **317** | |
| 022- | Baseline | 470 | 39 | 6.28x10$^{5}$ | 516 | 1.22 | **573** | | 435 | 584 | 0.59 | 810 | **329** | | | 500 | 1031 | 0.43 | **746** | |
| | Endpoint | 448 | 31 | 8.88x10$^{5}$ | 520 | 1.71 | **766** | | 431 | 592 | 0.46 | 824 | **296** | | | NOT CORONARY SINUS | | ..... | ..... | **Not Coronary Sinus ***** |
| 023- | Baseline | 259 | 38 | 7.62x10$^{5}$ | 318 | 2.4 | **623** | | 263 | 247 | 0.44 | 659 | **66** | | | 256 | 463 | 0.34 | **294** | |
| | Endpoint | 172 | 26 | 7.64x10$^{5}$ | 379 | 2.02 | **347** | | 175 | 170 | 0.59 | 410 | **40** | | | 166 | 259 | 0.85 | **234** | |
| 024- | Baseline | 340 | 17 | 6.58x10$^{5}$ | 388 | 1.7 | **578** | | 314 | 362 | 0.63 | 552 | **136** | | | 366 | 436 | 0.65 | **308** | |
| | Endpoint | 314 | 17 | 7.43x10$^{5}$ | 502 | 1.48 | **465** | | 323 | 342 | 0.85 | 326 | **96** | | | 305 | 367 | 0.64 | **257** | |
| 025- | Baseline | 370 | 100 | 5.71x10$^{5}$ | 304 | 1.88 | **696** | | 372 | 573 | 0.38 | 462 | **158** | | | 367 | 651 | 0.49 | **464** | |
| | Endpoint | 308 | 172 | 4.55x10$^{5}$ | 326 | 1.4 | **431** | | 315 | 589 | 0.59 | 389 | **107** | | | 300 | 530 | 0.5 | **380** | |
| 027- | Baseline | 258 | 85 | 4.13x10$^{5}$ | 465 | 0.89 | **230** | | 267 | 200 | 0.65 | 793 | **84** | | | 249 | 220 | 0.59 | **118** | |
| | Endpoint | 229 | 63 | 5.54x10$^{5}$ | 533 | 1.04 | **238** | | 217 | 365 | 0.65 | 645 | **186** | | | 238 | 321 | 0.59 | **223** | |
| 028- | Baseline | 286 | 112 | 4.22x10$^{5}$ | 240 | 1.76 | **501** | | 282 | 354 | 0.39 | 706 | **128** | | | 290 | 429 | 0.51 | **287** | |
| | Endpoint | 309 | 140 | 7.32x10$^{5}$ | 529 | 1.38 | **426** | | 304 | 412 | 0.65 | 655 | **200** | | | 313 | 497 | 0.52 | **347** | |
| 030- | Baseline | 400 | 98 | 4.22x10$^{5}$ | 331 | 1.27 | **508** | | 309 | 471 | 0.17 | 722 | **106** | | | 411 | 411 | 0.68 | **279** | |
| | Endpoint | 398 | 110 | 7.32x10$^{5}$ | 787 | 0.93 | **370** | | 357 | 433 | 0.49 | 722 | **181** | | | 440 | 454 | 0.65 | **300** | |
| 033- | Baseline | 430 | 150 | 3.79x10$^{5}$ | 661 | 0.57 | **245** | | 453 | 488 | 0.45 | 655 | **156** | | | 406 | 829 | 0.52 | **634** | |
| | Endpoint | 400 | 180 | 6.13x10$^{5}$ | Tracer | - | - | Extravas. | 431 | 590 | - | - | - | | | 369 | 741 | - | | **Tracer extravsation ***** |
| 034- | Baseline | 263 | 93 | 9.30x10$^{5}$ | 655 | 1.43 | **376** | | 275 | NOT RENAL VEIN | | ..... | **.....** | | | 251 | 372 | 0.3 | **196** | **Not renal \ Vein ***** |
| | Endpoint | 271 | 174 | 7.27x10$^{5}$ | 609 | 1.19 | **322** | | 245 | 277 | 0.37 | 542 | **66** | | | 296 | 371 | 0.6 | **253** | |
| 037- | Baseline | 250 | 73 | 10.07x10$^{5}$ | 769 | 1.31 | **328** | | 227 | 354 | 0.51 | 747 | **181** | | | 272 | 303 | 0.62 | **200** | |
| | Endpoint | 224 | 73 | 10.87x10$^{5}$ | 644 | 1.69 | **379** | | 200 | 325 | 0.56 | 750 | **178** | | | 247 | 404 | 0.72 | **335** | |
| 039- | Baseline | 219 | 83 | 9.35x10$^{5}$ | 700 | 1.34 | **293** | | 237 | 296 | 0.52 | 893 | **159** | | | 200 | 181 | 0.41 | **63** | |
| | Endpoint | 165 | 75 | 9.76x10$^{5}$ | 853 | 1.14 | **188** | | 165 | 348 | 0.62 | 647 | **185** | | | 165 | 158 | - | - | **Not Coronary Sinus ***** |
| 041- | Baseline | 242 | 151 | 10.77x10$^{5}$ | 585 | 1.84 | **445** | | 262 | 347 | 0.3 | 881 | **144** | | | 221 | 333 | 0.41 | **203** | |
| | Endpoint | 394 | 219 | 11.87x10$^{5}$ | 654 | 1.81 | **713** | | 339 | 405 | 0.56 | 739 | **174** | | | 450 | 480 | 0.53 | **269** | |
| 042- | Baseline | 333 | 97 | 15.92x10$^{5}$ | 913 | 1.74 | **580** | | 342 | 461 | 0.5 | 1010 | **293** | | | 323 | 408 | 0.64 | **292** | |
| | Endpoint | 310 | 196 | 11.22x10$^{5}$ | 609 | 1.84 | **570** | | 296 | 473 | 0.23 | 1130 | **277** | | | 342 | 349 | 0.75 | **264** | |
| 045- | Baseline | 527 | 110 | 6.55x10$^{5}$ | 820 | 0.8 | **422** | | 568 | 754 | 0.29 | 997 | **350** | | | 486 | 943 | 0.65 | **773** | |
| | Endpoint | 952 | 310 | 9.36x10$^{5}$ | 1003 | 0.93 | **885** | | 939 | 939 | 0.49 | 774 | **356** | | | 961 | 1443 | 0.43 | **895** | |
| 048- | Baseline | 298 | 234 | 8.9x10$^{5}$ | 681 | 1.31 | **390** | | 292 | 313 | 0.24 | 962 | **88** | | | 303 | 399 | 0.51 | **251** | |
| | Endpoint | 390 | 109 | 8.7x10$^{5}$ | 618 | 1.41 | **550** | | 488 | 375 | 0.14 | 1152 | **0** | | | 291 | 486 | 0.54 | **352** | |
| 049- | Baseline | 555 | 105 | 7.7x10$^{5}$ | 558 | 1.38 | **766** | | 606 | 733 | 0.15 | 1087 | **237** | | | 504 | 998 | 0.55 | **771** | |
| | Endpoint | 754 | 162 | 9.82x10$^{5}$ | 669 | 1.47 | **1108** | | 729 | 764 | 0.48 | 1118 | **428** | | | 779 | 740 | 0.68 | **491** | |
| 050- | Baseline | 512 | 213 | 7.6x10$^{5}$ | 727 | 1.05 | **538** | | 562 | 591 | 0.49 | 648 | **206** | | | 462 | 668 | 0.51 | **441** | |
| | - | | | | | | | | | | | | | | | | | | | **No Study Day 2 ***** |

LEGEND

Empagliflozin (yellow)

Placebo (green)
